# Minimal required PDT light dosimetry for nonmuscle invasive bladder cancer

**DOI:** 10.1117/1.JBO.25.6.068001

**Published:** 2020-06-11

**Authors:** Lothar Lilge, Jenny Wu, Yiwen Xu, Angelica Manalac, Daniel Molenhuis, Fynn Schwiegelshohn, Leonid Vesselov, Wayne Embree, Michael Nesbit, Vaughn Betz, Arkady Mandel, Michael A. S. Jewett, Girish S. Kulkarni

**Affiliations:** a University Health Network, Princess Margaret Cancer Centre, Toronto, Ontario, Canada; b University of Toronto, Department of Medical Biophysics, Toronto, Ontario, Canada; c University of Toronto, Department of Electrical and Computer Engineering, Toronto, Ontario, Canada; d Theralase Technologies Inc., Toronto, Ontario, Canada; e University of Toronto, Division of Urology, Department of Surgery, Toronto, Ontario, Canada

**Keywords:** TLD1433, irradiance sensor, FullMonte

## Abstract

**Significance:** Photodynamic therapy (PDT) could become a treatment option for nonmuscle invasive bladder cancer when the current high morbidity rate associated with red light PDT and variable PDT dose can be overcome through a combination of intravesical instillation of the photosensitizer and the use of green light creating a steep PDT dose gradient.

**Aim:** To determine how a high PDT selectivity can be maintained throughout the bladder wall considering other efficacy determining parameters, in particular, the average optical properties of the mucosal layer governing the fluence rate multiplication factor, as well as the bladder shape and the position of the emitter in relationship to the bladder wall.

**Approach:** We present three irradiance monitoring systems and evaluate their ability to enable selective bladder PDT considering previously determined photodynamic threshold values for the bladder cancer, mucosa and urothelium in a preclinical model, and the photosensitizer’s specific uptake ratio. Monte Carlo-based light propagation simulations performed for six human bladders at the time of therapy for a range of tissue optical properties. The performance of one irradiance sensing device in a clinical phase 1B trial is presented to underline the impact of irradiance monitoring, and it is compared to the Monte Carlo-derived dose surface histogram.

**Results:** Monte Carlo simulations showed that irradiance monitoring systems need to comprise at least three sensors. Light scattering inside the bladder void needs to be minimized to prevent increased heterogeneity of the irradiance. The dose surface histograms vary significantly depending on the bladder shape and bladder volume but are less dependent on tissue optical properties.

**Conclusions:** We demonstrate the need for adequate irradiance monitoring independent of a photosensitizer’s specific uptake ratio.

## 1 Introduction

The management of nonmuscle invasive bladder cancer (NMIBC) remains controversial.^1^ Variation in practice patterns is high, which contributes to the very high cost per case. Patients face cystectomy with well-documented morbidity when intravesical therapy, usually Bacillus Calmette Guerin (BCG), fails to control recurrent disease. A recent review by Kamat et al.^2^ stated that the recurrence rate following BCG immunotherapy is in the 33% to 42% range, with around 10% of patients progressing to higher stage disease muscle invasion during therapy. Recurrence patterns include patients who are unresponsive or intolerant to BCG.^2^ Various approaches to prolong the recurrence-free interval and, in particular, reduce early recurrences have been investigated, such as intravesical chemotherapy.^3^ However, benefits are limited, and it is widely recognized that there is an unmet need for other more effective treatments.^4^

Photofrin-mediated photodynamic therapy (PDT) was once considered an alternative for these patients but fell out of favor due to the high percentage of patients suffering persistent incontinence and significant voiding symptoms.^5^^,^^6^ Various approaches to minimize PDT-induced morbidity were proposed, including the generation of steep PDT dose gradients in the bladder wall using intravesical photosensitizer instillation^7^ and short wavelength activation^8^^,^^9^ or a repeat treatment strategy.^10^ Instillation intended to take advantage of the intact urothelium’s barrier function, which protects normal bladder tissue, including detrusor muscle, as well as to avoid sensitization of the vascular system. This was previously thought to be a key element in delayed PDT-induced morbidity.^8^^,^^11^^,^^12^ Green PDT activation wavelengths are four to five times more rapidly attenuated in biological tissues,^13^ including the bladder; hence, further protection of the muscle layer is anticipated.

Conversely, the reduced albedo of the bladder wall at green wavelength lowers the integrating sphere multiplication factor for bladder treatment adversely affecting the overall irradiation at the bladder surface.^14^ Repeat PDT treatments delivered fractionated and or metronomic showed promising initial results.^15^^,^^16^ To date, none of these approaches have been reported in a phase II study.

When considering these PDT morbidity reducing strategies, the overall PDT efficacy must be maintained with the ability to treat NMIBC up to a depth of 2 mm^17^ to the mucosal layer for stage T1 tumors, without damaging normal urothelium. Maintaining normal urothelium reduces the risk of discomfort or morbidity post-therapy. Hence, PDT efficacy will depend on controlling the irradiance of the bladder wall, which is subject to the bladder wall’s albedo and the overall shape of the bladder.

Ruthenium and other transition metal coordination complexes have shown favorable photochemical and photophysical properties for use as photosensitizers.^18^ They can act through energy transfer or charge transfer, are photostable, and often present with no-observed-adverse-effect level at high administered doses.^19^

We recently introduced TLC1433, a ruthenium coordination complex [Ru(II)${\left(4,4^{\prime }\text{-dimethyl-}2,2^{\prime }\text{-bipyridine}(\mathrm{dmb})\right)}^{2}$(2-(2′,2”:5”,2′″-terthiophene)-imidazo[4,5-f]$[1,10]\text{phenanthroline}){]}^{2+}$.^20^ This photosensitizer is water-soluble with rapid clearance from the blood ($T_{1/2}=44.4\;h$). Its quantum gaps for energy and charge-transfer are comparable, and in the absence of a reducing agent such as $O_{2}$, the photosensitizer switches from a type II to a type I reaction. Ruthenium coordination complexes have inherently high photostability and a reasonable absorption cross-section with $\varepsilon =3157\;M\;{\mathrm{cm}}^{-1}$ at 525 nm association with different serum proteins.^21^ TLC1433 is one member of a family of transition metal coordination complexes currently being developed for PDT.^22^^–^^25^

The drug uptake selectivity was previously determined to be $0.4\pm 0.06\;\mathrm{mg}\;{\mathrm{kg}}^{-1}$ in normal bladder comprising urothelium, mucosa, and muscle and $77\pm 18\;\mathrm{mg}\;{\mathrm{kg}}^{-1}$ in AY-27 rat bladder wall tumor. The resulting selective uptake ratio (SUR) is 192.^26^ It is reasonable to assume an equally big SUR for human cases as the urothelium is positively charged, causing an electrostatic repulsion of the photosensitizer. Using the AY-27 rat tumor model, the sensitivity of normal urothelium, muscle tissue, and the tumor was defined for TLD1433-mediated PDT employing the photodynamic threshold concept *in vivo*.^27^ The resulting threshold value estimates were as follows: $T_{\mathrm{AY}\text{-}27}<2.1210^{18}\;h\nu \;{\mathrm{cm}}^{-3}$, $T_{\text{urothelium}}>0.1610^{18}\;h\nu \;{\mathrm{cm}}^{-3}$, and $T_{\text{muscle}}>0.128\;10^{18}\;h\nu \;{\mathrm{cm}}^{-3}$. Hence, in the worst-case scenario, the AY-27 tumor cells would require ∼13 times higher TLD1433-generated reactive oxygen species (ROS) dose than normal urothelial cells. This is not unexpected as most tumors present with a higher ability to compensate for PDT-induced ROS, as previously seen, for example, in brain tumors.^28^ PDT selectivity in the mixed tumor normal tissue environment is given when Eq. (1) is satisfied throughout the bladder wall:

$$\frac{T_{\mathrm{AY}-27}}{{\left[\mathrm{TLD}1433\right]}_{\mathrm{AY}-27}}\frac{\varphi (0)}{\varphi (d)}<\frac{T_{\text{normal}}}{{\left[\mathrm{TLD}1433\right]}_{\text{normal}}}.$$ (1)

In the equation, normal denotes urothelium and muscle, [TLD1433] the measured tissue concentration, φ, the fluence (${J\cdot \mathrm{cm}}^{-2}$), and d (mm) the maximum desired treatment depth. $T_{\mathrm{AY}-27}$ and $T_{\text{normal}}$ are the PDT dose values, given in the number of photons absorbed per unit volume [$h\nu \;{\mathrm{cm}}^{-3}$] that needs to be exceeded for the tumor, but yielded for normal tissues. Satisfying the aforementioned condition, for all positions on and inside the bladder wall provides complete PDT selectivity. With an SUR of 192 (as previously reported), a target AY-27 tumor to normal urothelium threshold ratio of 0.0757, and considering a spherical shape $\frac{\varphi (0)}{\varphi (d)}∼14.5$, or it is allowing for 2.5 effective attenuation depths (${\mu_{\mathrm{eff}}}^{-1}$) to maintain PDT selectivity. Assuming an (${\mu_{\mathrm{eff}}}^{-1}$) of 750  μm for green wavelengths,^13^ a depth selectivity of 2 mm is feasible. Hence, if the tissue surface irradiance or radiant exposure can be well controlled, targeting recurrent and persistent NMIBC is possible. However, transurethral resection of bladder tumor (TURBT) is required for any larger visible tumors prior to PDT.

To maintain the PDT selectivity, control of the surface radiant exposure across the bladder wall surface is a prerequisite. However, as pointed out by Marijnissen et al.,^29^ Van Gemert et al.,^30^ and Van Stavern et al.^14^ and more recently by Rafailov et al.,^31^ the bladder is a hollow cavity acting as an integrating sphere, albeit with poorly defined optical properties. Thus, the bladder wall surface irradiance varies strongly with changes in the optical properties of the mucosa and the tumor, which govern the tissue albedo or backscattering power. For red wavelength and healthy bladders, multiplication factors of 6 were measured severely affecting the overall PDT delivered.^14^ For green light, smaller variations in the multiplication factors are anticipated, but knowledge of either the tissue optical properties or the actual irradiance inside the bladder cavity is required to maintain treatment selectivity across the bladder wall depth, particular as deviations from a spherical shape will introduce additional variations in the surface irradiance. Both the unknown multiplication factor and variations of bladder shape can result in an unknown fluence rate throughout the bladder wall, thereby limiting accurate prediction of the radiant exposure or total PDT dose delivered and thus PDT selectivity across the bladder wall.

Here, we present the developments and calibration of a new dosimetry system with up to −12 irradiance probes position and absolute irradiance measurements made in six human bladders during TLD1433-mediated PDT. The performance of the 12 positions irradiance sensing device is compared to two other possible designs comprising a single or three sensors based on Monte Carlo code-generated dose surface histogram (DSH) for a range of tissue optical properties. The FullMonte, Monte Carlo code was used to enable simulations of light propagation for irregular surfaces.^31^^,^^32^ The simulation DSHs were compared to measurements collected *in vivo*.

## 2 Materials and Methods

### 2.1 Irradiance Monitors Engineering and Calibrations

In this study, we considered three different designs for irradiance monitoring. The first implementation is a single position sensor at the bladder neck, permitting the use of a cut end fiber-based design, compatible with the working channel of a flexible cystoscope. The second implementation, comprised of a device with up to 12 irradiance monitoring probes placed in three lines along the bladder wall separated by 120 deg but necessitating the use of a ridged cystoscope due to the added space requirements. The third implementation comprises three cut-end optical fibers observing different surface areas of the bladder wall, after being polished at an angle of ∼25  deg. This design could also be suitable for a flexible cystoscope.

**Fig. 1:**
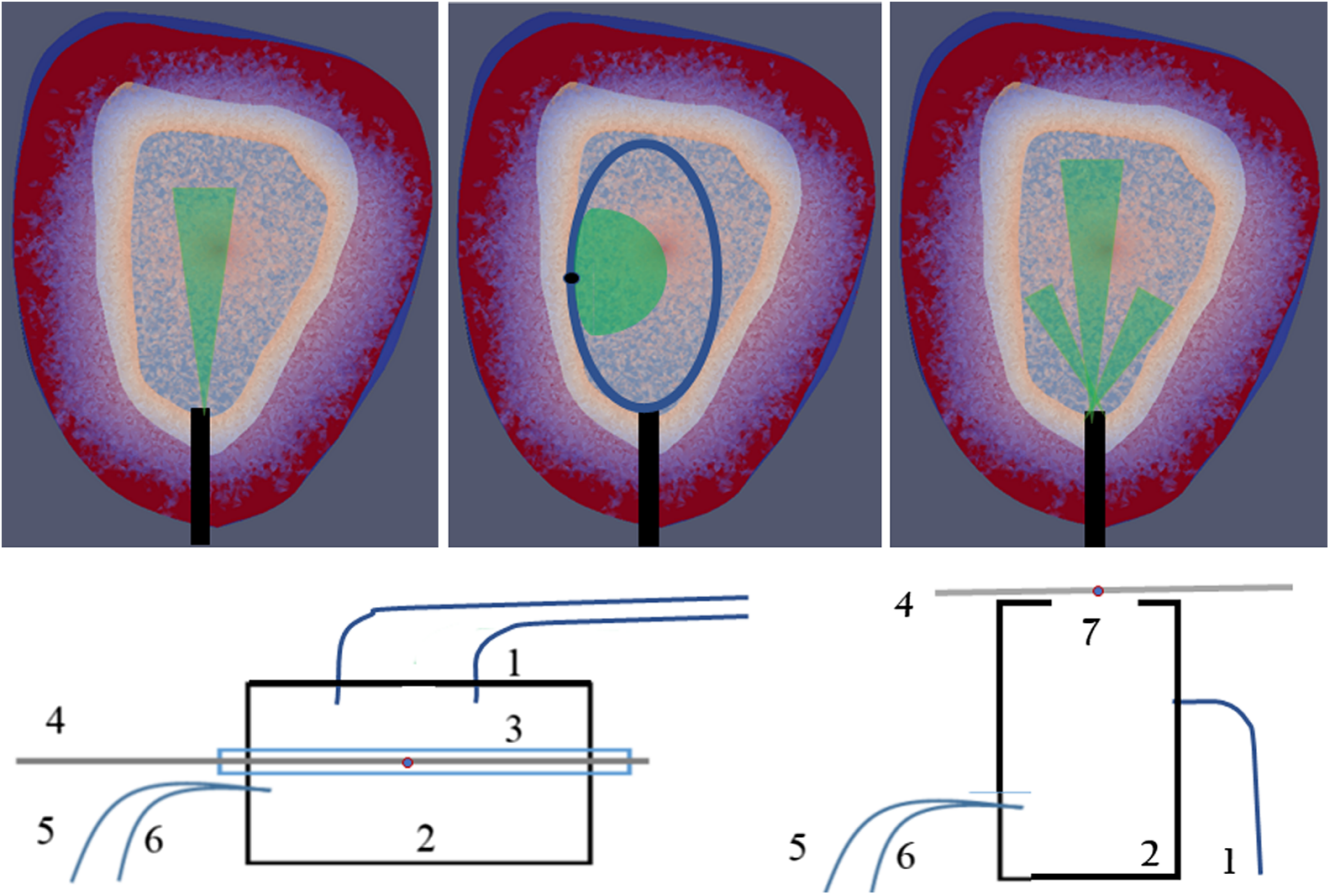
Top schematics of irradiance sensors design and indicating collection angles. (a) Single-sensor design, (b) 12-sensor design showing the acceptance angles for only one detector, (c) triple-sensor design. Bottom integrating spheres designs (d) irradiance sensor development and (e) irradiance sensor calibration. 1 indicates the illumination fibers, 2 represents the integrating cylinder, 3 the glass tube passing through the cylinder holding the POF 4, to a PIN diode. A reference fiber 5, is connected to a matched PIN diode (output in V), whereas reference fiber 6 is connected to an NIST calibrated power meter (output in W), 7 emission port providing 2π sr radiance exposure.

For the 12-sensor implementation, irradiance detectors were 250-μm-outer diameter plastique optical fibers (POFs) (SK-10, Mitsubishi Rayon Co. Ltd., Minato-ku, Japan). For implementations 1 and 3, the sensor is sensitive to the primary irradiance emitted by the source and diffuse reflected light from the opposite bladder wall, which satisfies the numerical aperture (NA) of the optical fibers, for the simulation assumed to be 0.625. For the second implementation, individual irradiance sensors were constructed by heating a 0.2-mm-wide metal blade to 75°C, the plastic’s transition temperature, and pressing it 75 to 100  μm into the POF at predetermined positions from the distal end of the fiber. The created depression is manually backfilled with barium sulfate doped medical grade epoxy (LOCTITE^®^M-31CL™, Henkel Loctite Americas, Quadra Chemicals, Oakville, Canada) to produce an isotropic fluence rate sensor accepting light over 4π sr. To reduce the responsivity to 2π sr, the backside of the sensor is blackened over a 1- to 2-mm long stretch. Four of these POaFs with different sensor positions, from their distal end, were aligned parallel and covered with a clear heat shrink tubing (103-0180, Nordson, Markham, Ontario, Canada) generating a flat fiber bundle. Three of these fiber bundles were linked at their distal end and rejoined into a single bundle 150 to 300 mm from the distal end, to accommodate different bladder sizes, and thus creating a cage with a central lumen for the isotropic light-emitting fiber. This assembly was fitted into a single sheath. The single and the triple sensor designs were also incorporated into the sheath of a catheter with a central lumen; however, extended <0.5  mm beyond the end of the catheter. For the third implementation, the optical fibers are polished at an angle of ∼25  deg and arranged within the sheath wall as for the single sensor design (Fig. 1).

For sensor development, an integrating cylinder was manufactured in house from a 200×100×100-mm ultrahigh-density polyurethane block (Gigahertz, Munich, Germany) with a 50-mm cylinder drilled out on the inside, as previously described.^32^ Two optical fibers connected to the laser source (ML6700, Modulight, Tampere, Finland) illuminated the cavity. Sensors were placed into the glass tubing during development, and their responsivity quantified by attaching the proximal end of the POF to an externally calibrated photodiode. (Fig. 1 Bottom left). For calibration, a 200-mm diameter integrating cylinder with a 25.4 mm emission port was used. The irradiance sensors were placed over the emission port and exposed to multiple irradiance settings to determine their responsivity (Fig. 1 Bottom right). Calibration was performed in air, and the refractive index change to the water-filled bladder required a correction factor of 1.58 as described.^33^^,^^34^

### 2.2 Light Source

The isotropic emitting light source (S85, Medlight SA, Switzerland) used for photosensitizer activation in the bladder comprised of a 0.85-mm emitter on a 400-μm low ${\mathrm{OH}}^{-1}$ silica fiber. The high NA of the fiber, 0.37, allows direct coupling to a laser without significant power losses. The fiber has a minimum bending radius of 47 mm, compatible with a flexible cystoscope, albeit that the cystoscopes bending radius at the distal end can be smaller, and care needs to be taken not to damage the optical fiber. In a liquid environment, a sustained power delivery of 2.5 to 2.8 W of 525-nm radiation was feasible without damage to the emitter.

### 2.3 In Silico Simulations

The ability of the three-sensor systems to monitor the average surface irradiance was modeled *in silico* based on the bladder shapes and volumes of six participants in a phase IB clinical trial^35^ of TLD1433-mediated PDT. Patients underwent a contrast-enhanced pelvic CT scan with 100 to 150 ml of Ultravist contrast medium (Bayer Inc., Mississauga, Ontario, Canada), providing a high-contrast image of the bladder using a Genesis CT scanner with 0.5-mm resolution (Canon Medical Systems Canada Limited, Markham, Ontario, Canada). The DICOM datasets were imported into ITK-SNAP,^33^ a threshold filter applied, and the surface contours were exported into a meshing tool (Meshing GUI developed in the group of Dr. V. Betz at the University of Toronto). The resulting tetrahedral-based three-dimensional image, comprising the bladder void and wall, and an optically thick layer of adipose tissue are compatible with Paraview’s .vtk file format (KitWare, Clifton Park, New York). Photon propagation simulations were executed using FullMonte.^34^^,^^36^ The photon absorption within the bladder wall, as well as the irradiance exiting the bladder void, was scored. The resulting local irradiance is superimposed on the bladder wall surface for illustration purposes or sorted by descending irradiance to achieve DSHs.

Tissue optical properties, at 525 nm, were $\mu_{a}=0.45\;{\mathrm{mm}}^{-1}$, $\mu_{s}=14.6\;{\mathrm{mm}}^{-1}$ in the bladder wall^13^ and $\mu_{a}=0.149\;{\mathrm{mm}}^{-1}$, $\mu_{s}=6.9\;{\mathrm{mm}}^{-1}$ in adipose tissue.^37^ The refractive index, n, and anisotropy factor, g, were set to 1.37 and 0.9 for both tissues. The bladder lumen was given optical properties of water: $\mu_{a}=0.000041\;{\mathrm{mm}}^{-1}$, $\mu_{s}=0.000017\;{\mathrm{mm}}^{-1}$, g=0.8, and n=1.33.^38^ Cystoscope observations of the bladder during short breaks in the PDT delivery showed on occasions turbidity within the bladder void, possibly caused by protein materials. To evaluate when scattering in the bladder volume also effected on the DSH, the $\mu_{s}$ for the water was increased up to $17\;{\mathrm{mm}}^{-1}$. Simulations were also carried out for a range of bladder wall tissue optical properties [$\mu_{a}$ and ${\mu_{s}}^{\prime }=\mu_{s}(1-g)$] and constant bladder void optical properties. Simulations required $10^{8}$ photon packets, requiring at most 14 min runtime on an Intel^®^ Xeon CPU E5 2650 V4@ 2.20 GHz with 24 CPU cores and 126 GB RAM when running 16 threads. The $10^{8}\;\mathrm{photon}$ packets were set to 2.5 W optical power delivered via the isotropic emitter placed either into the bladder’s geometric center or 1 cm more dorsally.

### 2.4 In Vivo Measurements of Irradiance in the Bladder

*In vivo* measurements were performed in six patients participating in a phase IB clinical trial to TLD1433 for NMIBC^35^ for the 12 sensors cage device. The positioning of the sensors was achieved by first placing the distal end of a ridged cystoscope at the bladder neck where the urethra starts. A sheath carrying the fiber bundle and isotropic emitting source was inserted through the cystoscope’s working channel, and the fiber bundle assembly extended until its distal end of the design touched the bladder’s dome. Pushing the three flat fiber bundles further through the working channel while keeping the cystoscope to bladder dome distance constant and pulling back on the control sting resulted in the expansion of the fiber cage until, ideally, the three arms abutted to the bladder wall. The proximal end of the fiber cage assembly was connected to a 12-channel photodiode array utilizing highly sensitive Hamamatsu detectors (S8745-1, Hamamatsu, Hamamatsu City, Japan) as the anticipated irradiances would generate only 80 nW to 4  μW optical power per sensors. Irradiances were recorded continuously and time-integrated to derive the radiant exposure. Excitation light delivery was terminated when the average of the responding sensors reached the target radiant exposure of $90\pm 9\;{J\cdot \mathrm{cm}}^{-2}$, shown in animal experiments to be a safe exposure resulting in tissue necrosis.^26^

## 3 Results

**Fig. 2:**
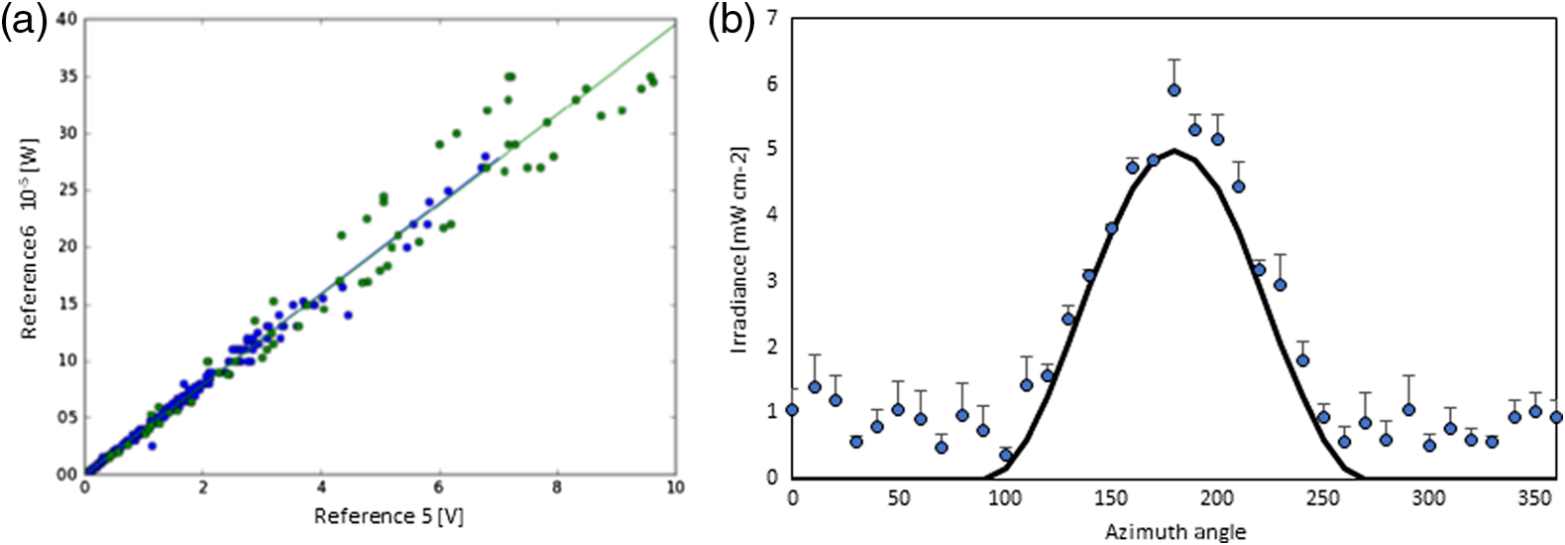
(a) Calibration data for an individual 2π sr irradiance sensor. Data are shown for two calibrations performed on separate days. (b) Averaged azimuth response of three black backside sensors indicating strong preferential detection from one hemisphere. The solid black line represents a $\cos{\left(\alpha \right)}^{2}$ responsivity, here only as illustration.

**Fig. 3:**
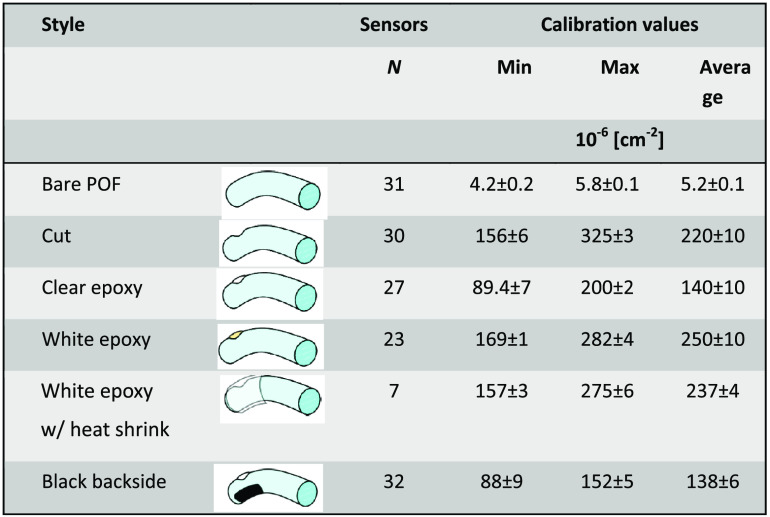
Calibration factors for irradiance sensors’ responsivity difference during production.

The solid detection angle of the 12 sensors implementation is two 2π sr or 6.28 sr, whereas the solid angle for irradiance sensors based on the cut end and polished optical fibers is only 0.187 sr given the aqueous environment. Testing the implementation 2 sensor’s response comprised a nonmodified POF without or with heat shrink tubing applied to demonstrate lack of light coupling along an unmodified fiber, a POF post applying the heated metal, post applying the epoxy with and without ${\mathrm{TiO}}_{s}$, and finally after applying the black nail polish. Figure 2 shows a typical calibration curve, and Fig. 3 shows the resulting responsivities for the modifications given in nW per ${\mathrm{mW}\cdot \mathrm{cm}}^{-2}$ or a sensor cross-section of $10^{-6}\;{\mathrm{cm}}^{-2}$. Three black backside sensors were placed into the center of a goniometer and the determined angular responsivity follows the $\cos{\left(\alpha \right)}^{2}$ law [Fig. 2(b)].

Applying the black backside to the white epoxy-filled cuts reduced the sensor’s responsivity by close to half, whereas the reduction of clear epoxy-filled cuts was <35% (data not shown) suggesting a directional response. Applying the heat shrink reduced a sensor’s responsivity only slightly but did not affect the azimuth response.

**Fig. 4:**
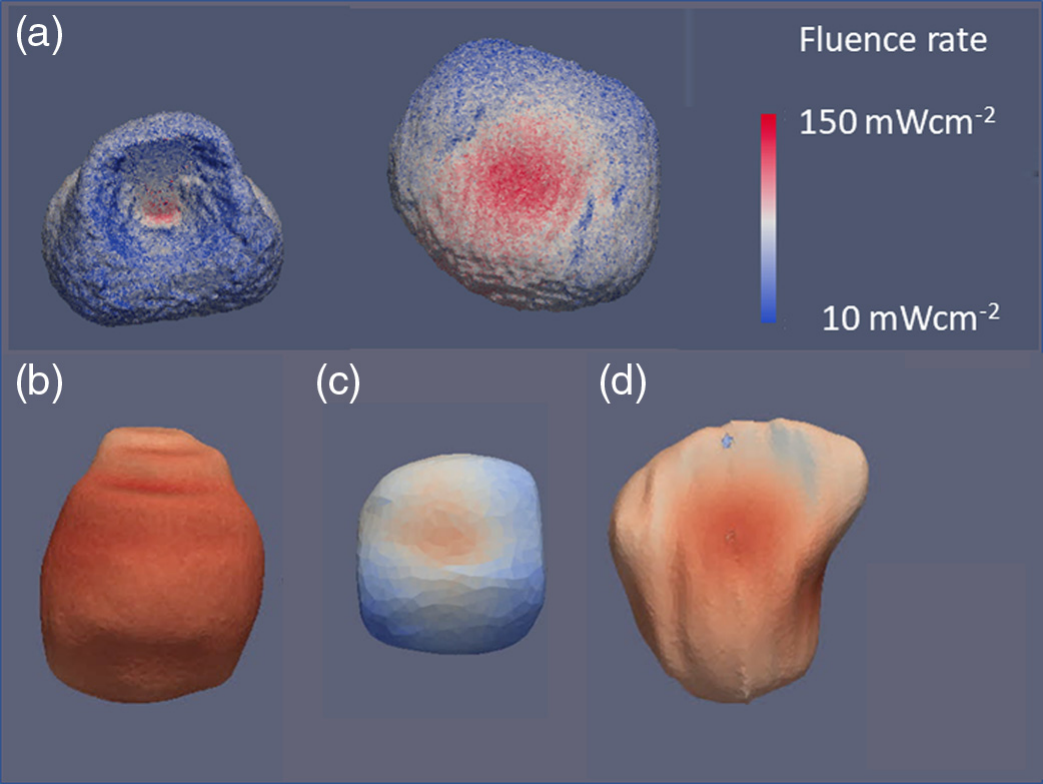
Visualization of the irradiance entering the bladder wall for 4 differently shaped and sized bladders. Optical properties assumed for the simulations as listed in the text. (a) The bladder contours of a male person, left transverse plane looking superior and right coronal plane looking dorsal. Note the deformation by the prostate in the transverse plane. (b) and (c) Transverse planes looking dorsal, (d) the sagittal plane. Fluence rates are given for a source power of 2.5 W at 525 nm.

**Fig. 5:**
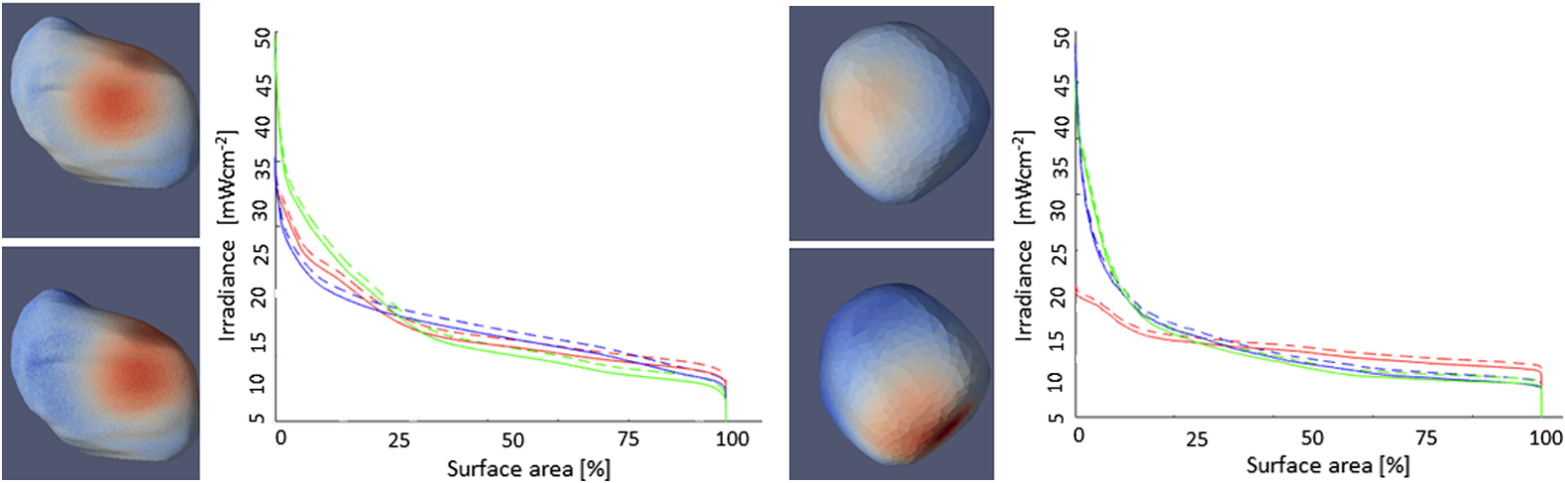
DSHs for two bladder contours. (a) Bladder volume 173 cc and (b) bladder volume 48.5 cc identical to Fig. 4(c). (Left) Void exiting irradiance for: (top) centrally placed emitter and (bottom) source displaced ventrally by 1 cm. (Right) The resulting DSHs. Red central, blue ventral displacement (+1  cm), and green dorsal displacement (−1  cm). Dashed lines for $\mu_{a}$ $0.45\;{\mathrm{mm}}^{-1}$ and solid lines for $\mu_{a}$ $0.675\;{\mathrm{mm}}^{-1}$.

Simulated irradiance for CT scans-based bladders shapes shows a variety of surface irradiance distributions (Fig. 4). The simulations are based on the tissue optical properties for adipose and bladder wall for 525 nm listed already. Large variations of the irradiance over the bladder void are visible due to the nonspherical bladder shape. A minor increase in the bladder wall absorption to $0.675\;{\mathrm{mm}}^{-1}$ equivalent to reducing the tissue albedo from 0.97 to 0.955 did not have a significant influence on the DSH (Fig. 5). Conversely, displacing the source, 1 cm from the geometric center of the bladder, increased the heterogeneity of the dose across the wall by up to a factor of 2 for bladder volumes <75  ml (Fig. 5).

**Fig. 6:**
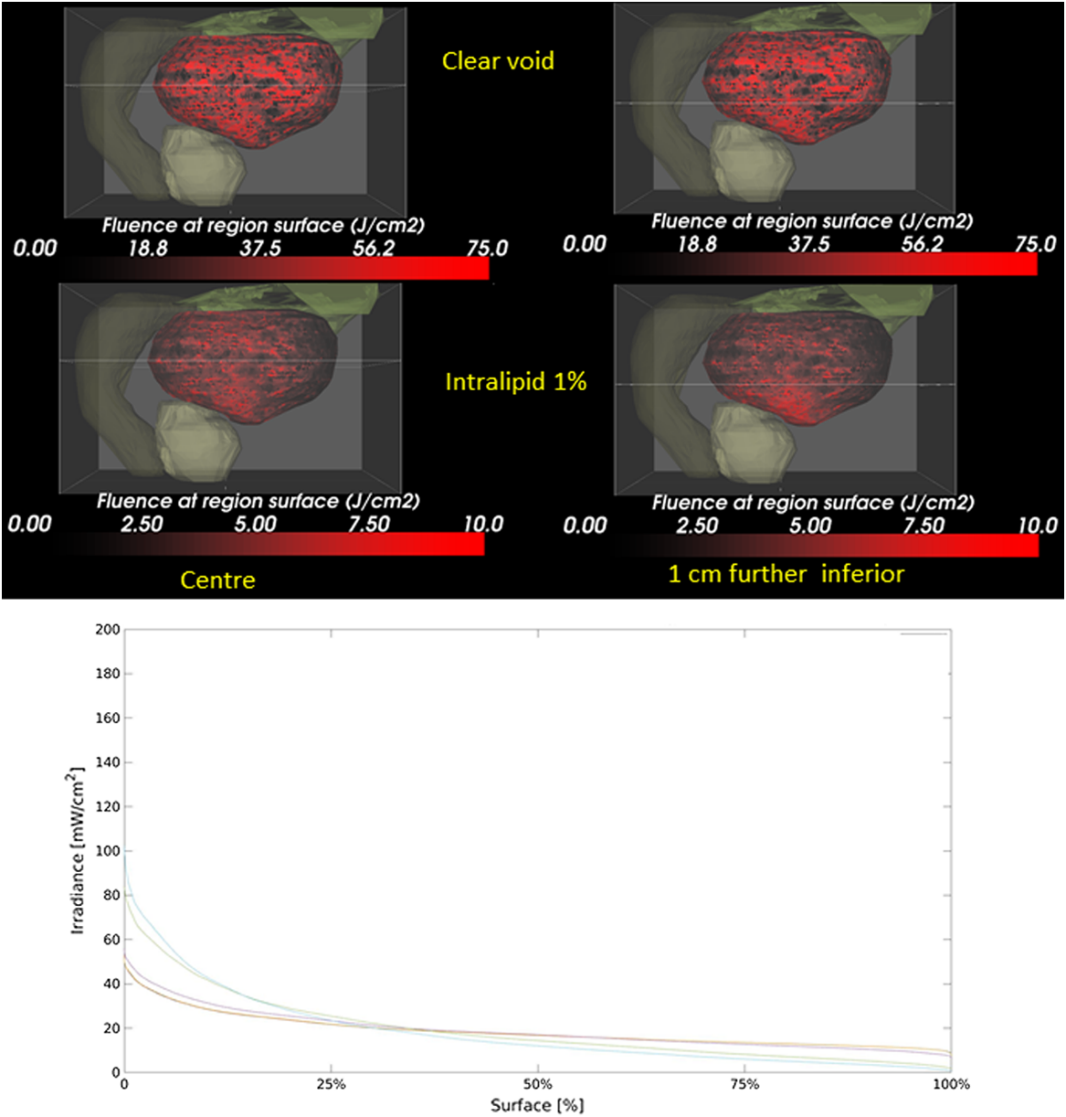
(a) Influence of light scattering in the bladder (${\mu_{s}}^{\prime }=87\;{\mathrm{mm}}^{-1}$) on the irradiance and homogeneity thereof on the bladder wall. Shown, the bladder (black and red), prostate green-gray), descending colon (dark green), and intestine (light green). (Left) Central positioned source, (Right) 1 cm below the central plane indicated by the horizontal line. (Top) Clear void and (bottom) assuming 1% intralipid in the bladder. Notice the hotspot at the bottom of the bladder in the lower right panel. (b) The graph shows the DSH for increasing the void’s scattering coefficient ${\mu_{s}}^{\prime }$ from 0.00017 to $17\;{\mathrm{mm}}^{-1}$.

Clinically, the position of the source was verified by ultrasound prior to the light treatment. A 1% intralipid solution, with ${\mu_{s}}^{\prime }=87\;{\mathrm{mm}}^{-1}$ and $\mu_{t}=460\;{\mathrm{mm}}^{-1}$,^39^^,^^40^ significantly reduces the irradiance on the bladder wall and augment the irradiance heterogeneity on the bladder wall surface following tissue misplacements by 1 cm (Fig. 6). Clinically, relevant deviations are observed in the DSHs only for $\mu_{s}\geq 1.7\;{\mathrm{mm}}^{-1}$.

**Fig. 7:**
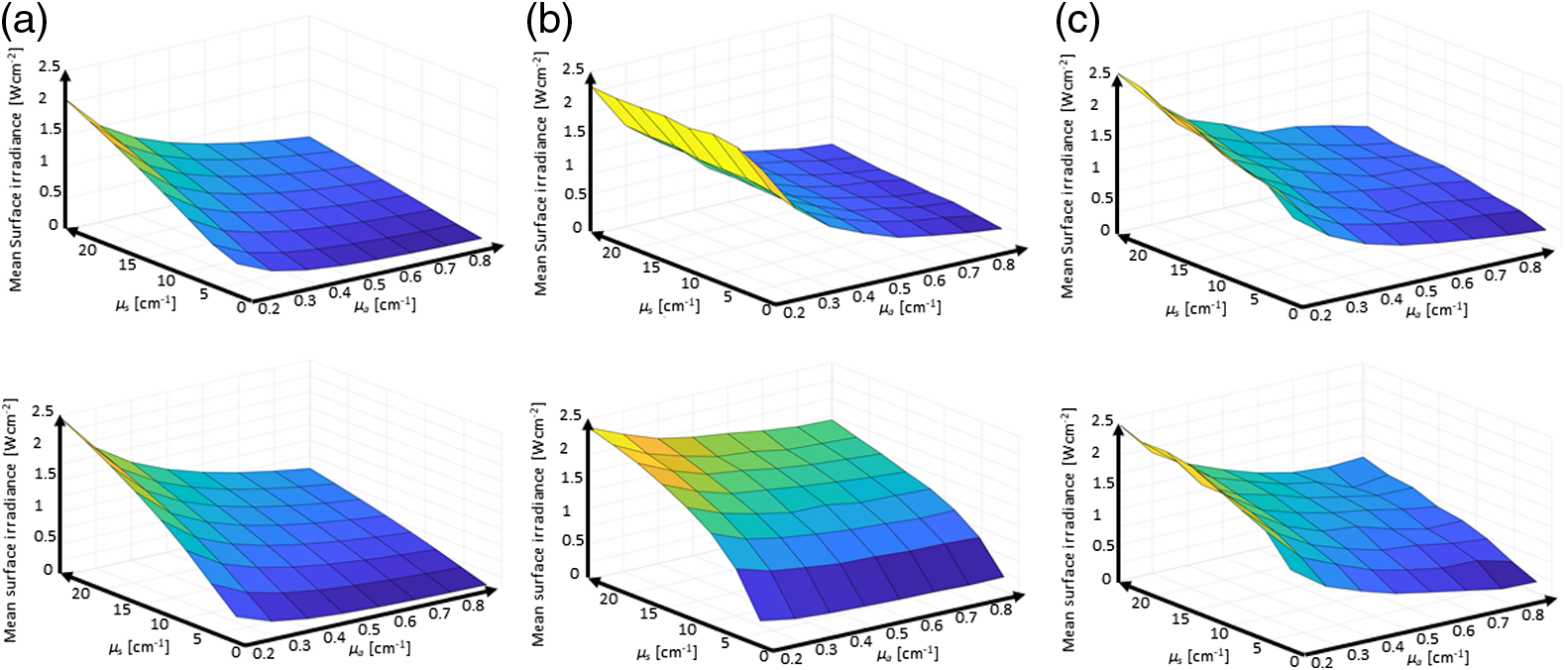
Monte Carlo simulation-based reporting of the mean bladder wall irradiance. Top row bladder C and bottom row bladder D from Fig. 2. (a) Mean irradiance on the bladder wall; (b) the average of the single sensor implementation; and (c) the average of the 12-sensors implementation.

**Fig. 8:**
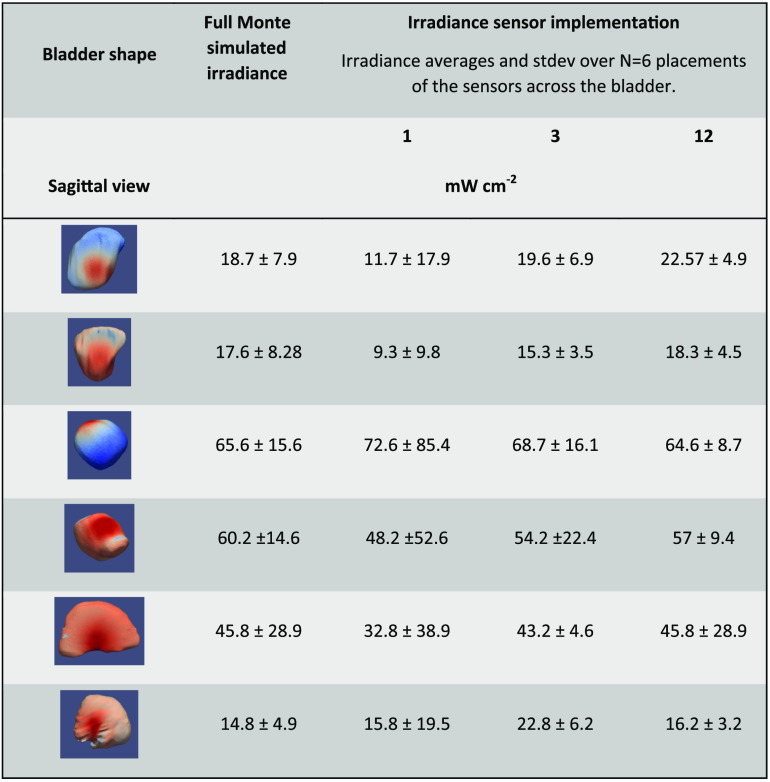
Range of the mean bladder wall irradiance to mean sensor reported irradiances ratios for the three implementations in the six investigated bladder shapes.

As the tissue optical properties in the bladder, around 525 nm, depend strongly on the tissue’s hemoglobin concentration, which in turn depends on the inflammation and extent of disease, the ability of the three sensor implementations to quantify the average tissue irradiances was evaluated as a function of the bladder wall’s tissue optical properties. The $\mu_{a}$ was varied from 0.2 to $0.9\;{\mathrm{mm}}^{-1}$ and $\mu_{s}$ from 0.5 to $25\;{\mathrm{mm}}^{-1}$ representing albedos from 0.035 to 0.992. Figure 7 shows the mean surface irradiance of a bladder as a function of the tissue optical properties, and the mean irradiance detected by the three sensor device implementations virtually placed on the surface. Figure 8 reports the average irradiance range for the different bladder shapes.

**Fig. 9:**
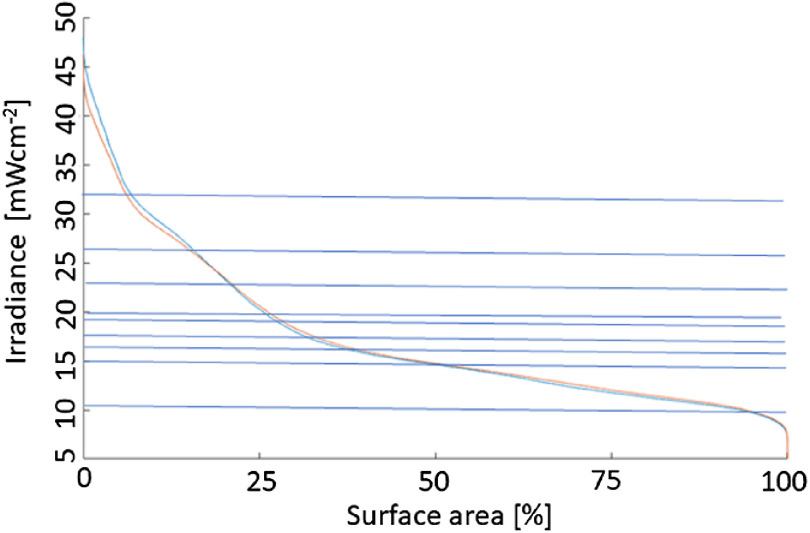
DSH based on Monte Carlo simulations. The nine horizontal lines indicate the irradiances measured during a clinical case at the onset of the procedure. The blue (top) curve at 0% surface area represents the source displaced by 1 cm from the center.

The advantage of using a large number of sensors is shown in Fig. 9, showing simulated DSHs for one of the bladder shapes with the source at the center and displaced by 1 cm dorsal. Superimposed are nine actual physical measurements in the bladder, showing that the dynamic range of the DSH can somewhat be captured, albeit the histogram’s actual shape would remain unknown.

**Fig. 10:**
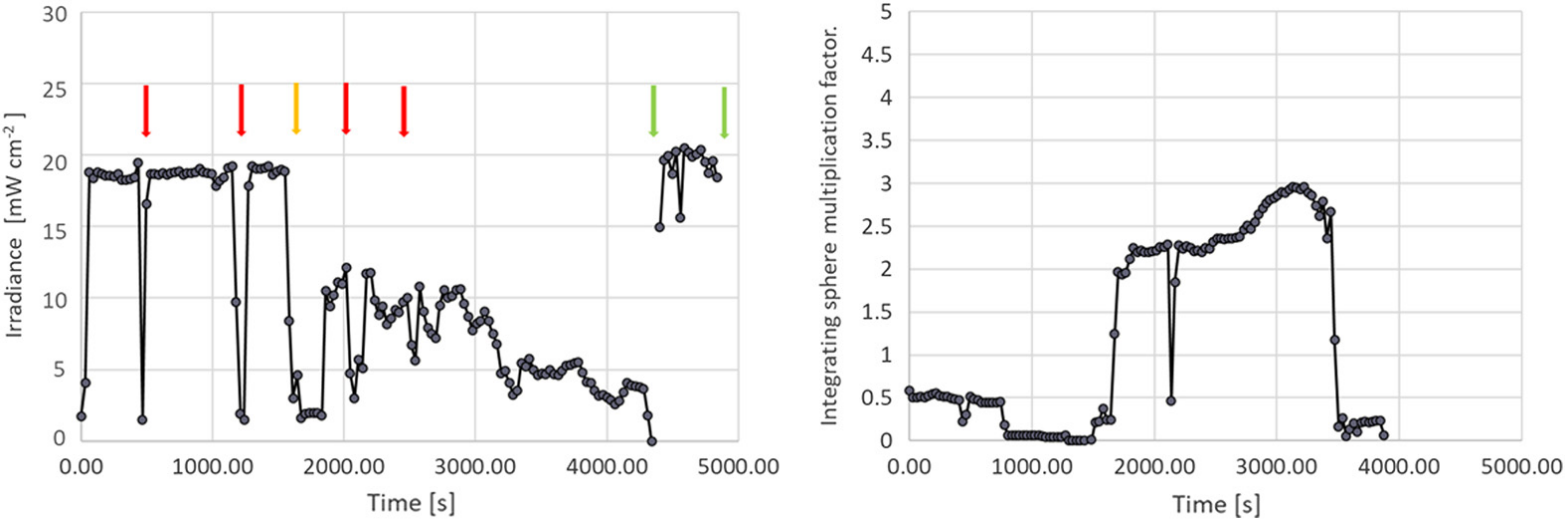
Average irradiance measured across eight sensors in a 173 cc large bladder. Red arrows indicate illumination interruptions when the urologist wanted to verify the position of the source within the bladder. The orange arrow indicated motion by the patient when repositioning of the source was required. The first green arrow indicates replacement of the emission fiber. The second green arrow termination of light exposure. The integral under the curve represents the surface radiant exposure. Note the positive offset at t=0 and various other times during treatment interruptions is due to illumination of the bladder by the cystoscope light.

Initial observations showed that the irradiance at the surface does not remain constant during the treatment. Drops in the irradiance were noted frequently (Fig. 10). Often due to the accumulation of protein materials around the warm light-emitting tip and light-scattering material accumulating in the bladder void.

## 4 Discussion and Conclusions

TURBT is the initial treatment for patients staged with NMIBC (stages at $T_{a}$, HG, $T_{1}$, and CIS), which is followed by BCG instillation for those at high risk of recurrence. Similarly, all visible tumor should be removed by TURBT prior to PDT. A 2- to 3-week time delay between TURBT and BCG is reasonable to apply also between TURBT and PDT.

The irradiance sensors exhibited the anticipated linear response. The responsivity of the white epoxy-filled sensors was approximately halved by application of the black backing, indicating a reduction in the angular responsivity from 4π sr to 2π sr. This was also seen in the goniometer measurements with a dominant sensor responsivity between 90 deg and 270 deg. The responsivities were in the order of $10^{-6}$ to $10^{-4}\;{\mathrm{cm}}^{-2}$ comparable to other published isotropic fiber-based sensors.^41^^–^^43^ While the calibration of the radiance sensing by cut end fibers is straightforward, they only captured a fraction of the total irradiance. Only 2.904% of the 2π solid angle was captured by a cut-end fiber, and as the angular distribution of the diffuse reflected light within nonspherical cavities is unknown, the accuracy of the irradiance sensors will be limited. The ∼2.9% of the bladder wall remission captured by these sensors represents only a small fraction of the DSH (Fig. 5), and hence one sensor can reflect a noticeable uncertainty versus the average irradiance within the bladder. A three-fibers sensor system will collect data for close to 9% of the surface area. However, the fraction of the irradiance captured will depend on the actual bladder shape.

The *in silico* simulation demonstrates the considerable variations in the surface irradiance for the six bladders investigated. Within each of the bladders, the irradiance can vary by up to a factor of 5 (Fig. 5). Displacement of the isotropic emitter in a transparent void has only limited effects on the DSHs for large bladders. Conversely, positioning of the source fiber becomes very important for bladder volumes <100  ml equal a radius of <∼3  cm, where a displacement of only 1 cm can result in a strong localized increase of the irradiance at the bladder wall proximal to the emitter.

Light scattering inside the bladder void, intentionally or unintentionally, needs to be avoided (Fig. 6). Light scattering exceeding $1.7\;{\mathrm{mm}}^{-1}$ causes weak confinement of the fluence rate around the source and leads to a noticeable reduction in the overall and average irradiance attainable on the bladder wall. Source misplacements from the void’s geometrical center increase in the irradiance variation on the bladder wall, creating hot and cold spots. The notion that light scattering inside the bladder can improve the homogeneity of the surface irradiation needs to be jettisoned.

Small variations in the bladder wall’s tissue optical properties, such as an increase in $\mu_{a}$ by 50%, did not result in a clinically significant modification of the DSHs or would result in altered patient management. However, more substantial variations in the bladder wall’s optical properties will vary the cavities’ fluence rate multiplication factors, which was shown to vary strongly between persons.^29^ For the investigated ranges of tissue optical properties, $\mu_{a}=0.2$ to $0.9\;{\mathrm{mm}}^{-1}$ and $\mu_{s}=0.5$ to $25\;{\mathrm{mm}}^{-1}$, which covered albedos from 0.035 to 0.992 resulted in a fivefold difference in the average irradiance in all bladder shapes, so sometimes up to a factor of 10. From Fig. 7, it is evident that the two implementations with 12 irradiance sensors, column C, capture the average irradiance, column A, better than a single irradiance sensor with a reduced solid acceptance angle for the excitation light, column B. The accuracy of the three-sensor device is closer to the 12-sensor device than the one-sensor device.

While the spacing between the 12 irradiance sensors is fixed, the absolute positioning of the irradiance sensors in a patient’s bladder is not determined, and thus the *in vivo* measured irradiance cannot be associated with a particular position in the DSH. While the 12-sensor device does not capture the full DSH shape, it is a good estimate of the irradiance DSH dynamic range (Fig. 9).

The 12- and 3-irradiance sensor implementation can provide information about increasing light scattering inside the bladder, causing the treatment to proceed too slowly, by an overall drop in the irradiance (Fig. 10). Rinsing the bladder and filling it with fresh water typically restore the irradiance to the surface. An added advantage of the multisensor implementations is their utility detecting local increases in the irradiance, which could point to source motion relative to the bladder wall. At emission powers of ˜2.5W or higher, thermal burns are likely if the emitter comes into contact with the bladder wall. Hence, irradiance monitoring also represents an integrated part of patient safety.

Increasing the PDT depth selectivity is feasible by modifications to the threshold values for which various approaches have been proposed, including hypothermia for ALA-induced PpIX,^44^ adjuvant chemotherapy^45^ or use of targeted nanoparticles,^46^ and may need to be considered if the SUR of the photosensitizer is significantly lower.

**Fig. 11:**
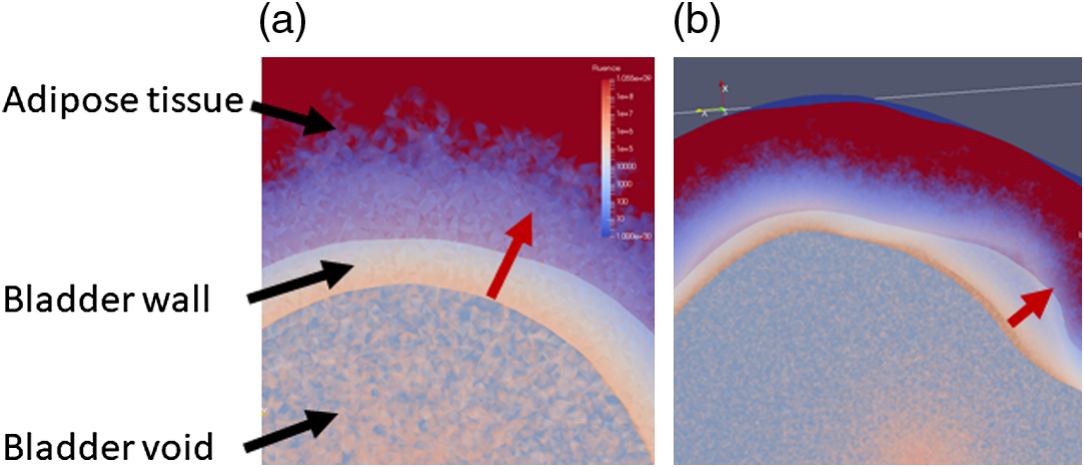
A detail look at the fluence rate distribution across the bladder wall. (a) A small, mostly spherical bladder [Fig. 4(c)] and (b) a nonspherical bladder [bladder in Fig. 4(d)]. The variations in the color-coded fluence rate in-depth based on the wall’s curvature are obvious as indicated along with the red.

In conclusion, the preclinical studies^26^ showed that TLD-1433 for NMIBC presents with a considerable SUR of 192. PDT treatment selectivity needs to ensure the threshold for the tumor being exceeded across the bladder wall but not for the normal urothelium. The worst-case estimate for the threshold values reported^26^ one that needs 13.2 times more photons to be absorbed in the tumor compared to the urothelium and detrusor muscle, respectively, to achieve selectivity. Selectivity between the tumor and the muscle is independent of light attenuation across the bladder mucosa, whereas selectivity between tumor and urothelium needs to consider the differences in fluence across the mucosa. Considering a maximum treatment depth of 2 mm, equivalent to $2.5\;{\mu_{\mathrm{eff}}}^{-1}$ at 525 nm, is feasible given the SUR and the worst-case tissue responsivity difference if a predetermined homogeneous radiant exposure can be delivered across the entire surface. As the absolute irradiance or radiant exposure varies up to a factor of 5, depending on the bladder wall’s optical properties, irradiance monitoring throughout the light exposure is needed. Irradiance monitoring cannot compensate for the variations in the DSHs seen due to the bladder’s shape. However, the range of irradiances in the histograms appears to be limited in general up to a factor of 5. One caveat is the assumption that the fluence at depth is only determined by the total radiant exposure at the surface; however, as Fig. 11 demonstrates, the actual curvature of the bladder wall may have an impact on the fluence depth distribution. To estimate the influence of the bladder wall’s curvature, additional Monte Carlo simulations may be required.

Overall based on the preclinical studies compiled here, and the proof of concept of the irradiance monitoring system *in vivo* enables proceeding to the next clinical studies.
