# Minimal required PDT light dosimetry for nonmuscle invasive bladder cancer (Erratum)

**DOI:** 10.1117/1.JBO.25.6.069801

**Published:** 2020-06-26

**Authors:** Lothar Lilge, Jenny Wu, Yiwen Xu, Angelica Manalac, Daniel Molenhuis, Fynn Schwiegelshohn, Leonid Vesselov, Wayne Embree, Michael Nesbit, Vaughn Betz, Arkady Mandel, Michael A. S. Jewett, Girish S. Kulkarni

**Affiliations:** a University Health Network, Princess Margaret Cancer Centre, Toronto, Ontario, Canada; b University of Toronto, Department of Medical Biophysics, Toronto, Ontario, Canada; c University of Toronto, Department of Electrical and Computer Engineering, Toronto, Ontario, Canada; d Theralase Technologies Inc., Toronto, Ontario, Canada; e University of Toronto, Division of Urology, Department of Surgery, Toronto, Ontario, Canada

## Abstract

The erratum corrects a misspelled author surname.

This article [*J. Biomed. Opt.* **25**(06), 068001 (2020) doi: 10.1117/1.JBO.25.6.068001] was originally published online on 11 June 2020 with an error in the spelling of an author’s surname. The correct spelling is Molenhuis.

This article was corrected online on 16 June 2020.

